# Autophagy Is Rapidly Induced by Salt Stress and Is Required for Salt Tolerance in Arabidopsis

**DOI:** 10.3389/fpls.2017.01459

**Published:** 2017-08-22

**Authors:** Liming Luo, Pingping Zhang, Ruihai Zhu, Jing Fu, Jing Su, Jing Zheng, Ziyue Wang, Dan Wang, Qingqiu Gong

**Affiliations:** Tianjin Key Laboratory of Protein Sciences, Department of Plant Biology and Ecology, College of Life Sciences, Nankai University Tianjin, China

**Keywords:** autophagy, Arabidopsis, salt stress, autophagic flux, ATG8, NBR1, vacuoles

## Abstract

Salinity stress challenges agriculture and food security globally. Upon salt stress, plant growth slows down, nutrients are recycled, osmolytes are produced, and reallocation of Na^+^ takes place. Since autophagy is a high-throughput degradation pathway that contributes to nutrient remobilization in plants, we explored the involvement of autophagic flux in salt stress response of Arabidopsis with various approaches. Confocal microscopy of GFP-ATG8a in transgenic Arabidopsis showed that autophagosome formation is induced shortly after salt treatment. Immunoblotting of ATG8s and the autophagy receptor NBR1 confirmed that the level of autophagy peaks within 30 min of salt stress, and then settles to a new homeostasis in Arabidopsis. Such an induction is absent in mutants defective in autophagy. Within 3 h of salt treatment, accumulation of oxidized proteins is alleviated in the wild-type; however, such a reduction is not seen in *atg2* or *atg7*. Consistently, the Arabidopsis *atg* mutants are hypersensitive to both salt and osmotic stresses, and plants overexpressing ATG8 perform better than the wild-type in germination assays. Quantification of compatible osmolytes further confirmed that the autophagic flux contributes to salt stress adaptation. Imaging of intracellular Na^+^ revealed that autophagy is required for Na^+^ sequestration in the central vacuole of root cortex cells following salt treatment. These data suggest that rapid protein turnover through autophagy is a prerequisite for salt stress tolerance in Arabidopsis.

## Introduction

Soil salinity is a major abiotic factor that limits crop yield (Flowers, 2004; Munns and Tester, 2008; Hanin et al., 2016). Globally, about 900 million hectares of land were estimated to be saline, and more than 30% of the irrigated crops were salt-affected (FAO^1^) (Flowers, 2004; Schroeder et al., 2013). High concentrations of NaCl inhibits plant water uptake, and Na^+^ and Cl^-^ accumulated in the cytosol lead to ion toxicity. As results, photosynthetic rates are reduced, leading to energy depletion and accumulation of excessive reactive oxygen species (ROS) (Zhu, 2002; Deinlein et al., 2014; Golldack et al., 2014; Julkowska and Testerink, 2015). In order to alleviate the osmotic and ionic stresses, plants close their stomata to minimize water loss (Munemasa et al., 2015), reduce their growth rates (Julkowska and Testerink, 2015), and limit intracellular Na^+^ concentration by compartmentalization in the vacuole (Munns and Tester, 2008). Gradually, relocation of Na^+^ away from young tissues takes place to exclude Na^+^ from growing organs (Munns and Tester, 2008; Deinlein et al., 2014), and plants may enter a growth recovery phase to resume growth at a reduced steady rate (Munns, 2002; Julkowska and Testerink, 2015).

With engineering of salt-tolerant crops in mind, numerous studies have been carried out to elucidate salt stress response, with emphases on transcription regulation and ABA signaling (Urano et al., 2010; Osakabe et al., 2014). Enormous progress have also been made in understanding the ion transport mechanisms, such as the discovery of Salt Overly Sensitive (SOS) signaling cascade and the in-depth studies on the HKT and NHX transporters (Qiu, 2012; Ji et al., 2013; Volkov, 2015). Additionally, the molecular basis for tissue tolerance has been revealed, which includes biosynthesis of osmolytes, such as glycinebetaine, sugar alcohols, polyamines, and proline (Tarczynski et al., 1993; Szabados and Savoure, 2010; Chen and Murata, 2011).

Compared with the topics above, how salt-challenged plants manage to maintain their energy level and re-allocate the limited resources is less well understood. One pathway that may contribute to salt-elicited nutrient recycling is macroautophagy (hereafter autophagy) (Han et al., 2011; Li and Vierstra, 2012; Liu and Bassham, 2012).

Autophagy is a bulk degradation pathway that helps maintain cellular homeostasis (He and Klionsky, 2009; Mizushima et al., 2011; Liu and Bassham, 2012; Ohsumi, 2014). In this pathway, obsolete proteins and damaged organelles are enveloped by an expanding double-membraned vesicle, the isolation membrane/phagophore, which matures to a sealed autophagosome before fusing with the lytic vacuole (Li and Vierstra, 2012; Lamb et al., 2013; Shibutani and Yoshimori, 2014; Michaeli et al., 2016; Zhuang et al., 2016). The inner membrane of the autophagosome along with the cargo, termed the autophagic body, is then degraded by vacuolar hydrolases, and amino acids and other macro molecules are released back into the cytosol through transporters (Kuma and Mizushima, 2010; Shibutani and Yoshimori, 2014).

The hallmark of autophagy is the formation of the autophagosome (Xie and Klionsky, 2007; Shibutani and Yoshimori, 2014; Zhuang et al., 2016). As key players in this process, autophagy-specific ubiquitin-like proteins (UBLs) ATG8/LC3/GABARAP act as protein scaffolds to mediate phagophore expansion (Nakatogawa et al., 2007; Xie et al., 2008; Weidberg et al., 2010). Conjugation of ATG8 to the phagophore requires the activity of several other ATG proteins (Shibutani and Yoshimori, 2014). Firstly, newly synthesized ATG8 is truncated by the cysteine protease ATG4 to expose the C-terminal Glycine residue (Kirisako et al., 2000; Woo et al., 2014). The Glycine is then conjugated to the amino group of Phosphatidylethanolamine (PE) in a ubiquitin-like conjugation reaction catalyzed by ATG7 as the E1, ATG3 as the E2 (Ichimura et al., 2000), and the ATG12-ATG5-ATG16 complex as the E3 enzyme (Hanada et al., 2007). PE-conjugated ATG8 stably associates with both phagophore and completed autophagosomes, hence is commonly used as a marker for microscopic study of autophagy (Kabeya et al., 2000; Yoshimoto et al., 2004; Contento et al., 2005). In addition, PE-conjugated ATG8 moves faster than the unconjugated ATG8 in SDS-PAGE gels, hence the amount of lipidated ATG8 is indicative of autophagic activity (Kabeya et al., 2000). Furthermore, by comparing the ATG8-PE levels in the presence and absence of vacuolar protease inhibitors, such as E-64d, or tonoplast H^+^-ATPase inhibitors, such as concanamycin A (Con A), autophagic flux can be quantified (Mizushima and Yoshimori, 2007). The selective autophagic flux can be detected by measuring Neighbor of BRCA1 (NBR1) degradation (Mizushima and Yoshimori, 2007; Svenning et al., 2011). During selective autophagy, NBR1 binds both ATG8 and mono- and (especially) poly-ubiquitin, linking the ubiquitinated cargoes to the autophagy machinery. Then it is transported together with the cargoes inside the autophagosome to the lytic vacuole for degradation (Mizushima and Yoshimori, 2007; Svenning et al., 2011). Hence NBR1 serves as both a receptor and a selective substrate of autophagy, and the degradation of NBR1 is indicative of selective autophagic flux (Mizushima and Yoshimori, 2007). Therefore, immunoblotting with antibodies against ATG8, NBR1, or epitope tags fused to ATG proteins provides more quantitative information for detecting autophagy (Bao et al., 2016).

To see if the level of autophagic flux correlates with salt tolerance, Arabidopsis mutants defective in autophagy and mutants with reduced level of autophagy, as well as transgenic plants that have increased level of autophagy were selected for further analyses (**Supplementary Figure S1**). The five ATGs belong to the core autophagy machinery (Xie and Klionsky, 2007). ATG5, ATG7, and ATG10 are required for ATG8-lipid adduct and autophagosome formation, and ATG5 have been shown to localize at the outer surface of the cortical endoplasmic reticulum (ER) to recruit ATG8 for phagophore assembly and expansion (Le Bars et al., 2014). Both *atg5* and *atg7* are autophagy-deficient mutants (Thompson et al., 2005; Inoue et al., 2006; Shin et al., 2014). ATG2 supposedly forms a complex with ATG18 to mediate the shuttle of ATG9 vesicles, which are a source of autophagosomal membranes (Yamamoto et al., 2012). Autophagic flux is known to be reduced rather than absent in *atg9* (Inoue et al., 2006; Shin et al., 2014; Zhuang et al., 2017). Transgenic Arabidopsis over-expressing GmATG8c (*ATG8-OX*) was used to represent plants with high levels of autophagy (Xia et al., 2012).

As a house-keeping pathway, autophagy is generally maintained as a basal level and can be quickly induced by nutrient deprivation and various stresses (He and Klionsky, 2009). Several lines of evidence suggest that autophagy is positively involved in plant salt stress adaptation. NaCl treatments have been shown to induce transcriptions of several *Autophagy-related (ATG)* genes, especially *ATG8*s and *ATG18*s in Arabidopsis, salt cress, rice, wheat, tobacco, pepper, and foxtail millet (Gong et al., 2005; Liu et al., 2009; Xia et al., 2011; Pei et al., 2014; Zhou et al., 2015; Li et al., 2016; Zhai et al., 2016). In Arabidopsis, *RNAi-AtATG18a* plants were hypersensitive to salt and osmotic stress in germination and seedling growth (Liu et al., 2009). The autophagy-deficient mutants, *atg5* and *atg7*, as well as the ATG8-interacting autophagy cargo adaptor mutant *nbr1*, exhibited sensitivity toward drought and salt treatments (Zhou et al., 2013). Mutation in *ATG8-Interacting Protein 1(ATI1)* led to salt sensitivity during germination (Michaeli et al., 2014). Intriguingly, it was reported that over-expression of *GFP–AtAtg8f-HA* led to reduced tolerance toward mild osmotic and salt stresses, but not to stronger stresses (Slavikova et al., 2008).

Many questions remain on how autophagy contributes to salt stress tolerance in plants. When does autophagic flux peak following salt stress? Does autophagy participate in the clearance of salt-induced oxidized proteins? Does it contribute to osmolyte production? Does it have a role in Na^+^ uptake or sequestration in the vacuole?

Here we show that the autophagic flux is rapidly induced by salt treatment. Then autophagy deficient (*atg5* and *atg7, atg10*), defective (*atg9* and possibly also *atg2*), and enhanced (*Pro35S:GmATG8c, ATG8-OX*) lines were selected to see if the levels of autophagy correlate with salt stress tolerance. Physiological analyses revealed that the *atg* mutants accumulated less soluble sugars and proline, whereas *ATG8-OX* plants accumulated more osmolytes. Oxidized protein is alleviated after treated with NaCl in the wild-type within 3 h. We also show that Na^+^ sequestration in the lytic vacuole of salt-stressed root cortex cells is correlated with the level of autophagy. Our observations suggest that salt stress rapidly triggers autophagy to facilitate bulk protein turnover, thus providing macromolecules and energy required for plant survival.

## Materials and Methods

### Accession Numbers

ATG2, At3g19190; ATG5, At5g17290; ATG7, At5g45900; ATG8a, AT4G21980; ATG9, At2g31260; ATG10, AT3G07525; NBR1, AT4G24690.

### Plant Materials and Growth Conditions

Arabidopsis (ecotype Columbia-0) was grown as described (Xiong et al., 2016). Generally, seeds were surface-sterilized with 75% ethanol for 5 min, 100% ethanol for 1 min, rinsed with ddH_2_O for five times, then stratified at 4°C for 2 days before plated on 1/2 Murashige and Skoog (1/2 MS) medium (Sigma–Aldrich, United States) containing 0.8% (w/v) agar (Sigma–Aldrich, United States), 1% (w/v) sucrose (Sigma–Aldrich, United States), pH5.7. Liquid 1/2 MS medium was prepared in the same way, only without agar. The plants were grown at 16 h (22°C)/8 h (18°C) with a photosynthetic photon flux density at 90 μE m^-2^ sec^-1^. The T-DNA insertion mutants *atg2-1* (Salk_076727), *atg5-1* (SAIL_129B07, CS806267), *atg7* (SAIL_11H07, CS862226), *atg9* (SAIL_527_A02, CS874564), and *atg10-1* (Salk_084434) were obtained from ABRC (Sessions et al., 2002; Alonso et al., 2003), and transgenic lines carrying *Pro35S:GmATG8c* were as described (Xia et al., 2012). All mutants and transgenic plants were verified by genomic PCR. Primers used are listed in **Supplementary Table S1**. All lines have been freshly propagated to ensure wild-type level germination rates on control medium. Phenotypes were documented as described (Xiong et al., 2016). All images were analyzed with Image J^2^, and statistical analyses (*F*-test, Student’s *t*-test) were done with Microsoft Excel 2010.

### Generation of an Autophagic Marker, *ProATG8a:GFP-ATG8a*

To construct *ProATG8a:GFP-ATG8a*, an 1199 bp fragment upstream of the start codon (ATG) of *ATG8a* (-1199 To 1) was PCR-amplified and inserted between *Pst* I and *Nco* I of *pCAMBIA1302*. Then a genomic fragment of *ATG8a* (942 bp) was inserted after GFP by homologous recombination with a ClonExpress II One Step Cloning Kit (Vazyme, Nanjing, China). The construct was verified by sequencing before introduced into *Agrobacterium tumefaciens* (GV3101) for floral dipping (Clough and Bent, 1998). Primary transformants were selected by antibiotic resistance and verified by PCR. The T3 homozygous marker line L5-1 was introduced into *atg10* by crossing, and *atg10*/*GFP-ATG8a* plants were identified by genotyping of F2 individuals.

### Immunoblotting

To quantify autophagic flux, 10-day-old vertically grown seedlings (approximately 200 mg) were transferred to liquid 1/2 MS medium with 150 mM NaCl for 0, 0.5, 1, 3, and 6 h before harvested. To block autophagosome degradation in the lytic vacuole, a parallel set of seedlings were transferred to medium containing NaCl plus 0.5 μM Concanamycin A (ConA, Sigma–Aldrich, United States) and harvested at the same time points. Protein extraction, quantification, and immunoblotting were done as described (Xia et al., 2012). All SDS-PAGE gels were prepared with 6 M urea. Primary antibodies used were anti-GmATG8c (1:3000) (Xia et al., 2012), anti-NBR1 (Agrisera, 1:3000), and anti-tubulin (Utibody, 1:5000). Each experiment was repeated for at least three times, and one representative result was shown. Quantification of immunoblots was done with Image J and statistical analyses (*F*-test, Student’s *t*-test) were done with Microsoft Excel 2013.

### Oxidized Protein Analysis

Oxidized protein analysis was performed following a previous report (Xiong et al., 2007). Ten-day-old seedlings grown on 1/2 MS vertical plates were transferred to liquid 1/2 MS medium with 150 mM NaCl for 0, 0.5, 1, 3, and 6 h before harvested for protein extraction. The extraction buffer contains 0.1 M Tris-HCl, pH 7.5, 0.3 M Sucrose, 1 mM EDTA, 0.1 mM phenylmethylsulphonylfluoride (PMSF), and 1% [v/v] β-mercaptoethanol. Extracts were centrifuged at 1,000 *g* for 10 min and supernatants were collected. Oxidized proteins were detected using an OxyBlot protein oxidation detection kit (Abcam, United Kingdom) according to the manufacturer’s instructions. Dinitrophenylhydrazine (DNP) signals (entire lane) were quantified by densitometry in Image J and normalized to the wild-type 0 h control value, which was set as 1. Each experiment was repeated for at least three times, and one representative result was shown. Coomassie blue staining of total proteins was used as the loading control.

### Quantification of Proline, Total Soluble Sugar, and Reducing Sugar content

Ten-day-old seedlings grown on 1/2 MS vertical plates were transferred to liquid 1/2 MS containing 150 mM NaCl for 0, 8, and 24 h. Harvested samples were weighed, and free proline was quantified with ninhydrin assay at A520 nm with a kit (Comin Biotech, Suzhou, China) following manufacturers’ instructions. Total soluble sugar and reducing sugar contents were determined by the anthrone reagent method and dinitrosalicylic acid (DNS) method with a kit (Yuanye Biotech, Shanghai, China) following manufacturers’ instructions. The sugar contents were determined against a standard curve prepared with glucose (Sigma–Aldrich, United States). Three biological replicates were done in each case with consistent results, and representative results are shown.

### Amino Acid Analysis

Ten-day-old seedlings grown on 1/2 MS vertical plates were transferred to liquid 1/2 MS containing 150 mM NaCl for 0, 8, and 24 h. A parallel set of seedlings were treated with NaCl plus 10 mM Chloroquine. Harvested samples were weighed and quantified for soluble amino acid on a membraPure Amino Acid Analyzer A300 (Germany) following manufacturer’s instructions.

### Stress Treatments

For germination assay, stratified seeds were sown on 1/2 MS medium with or without NaCl or mannitol. Four biological replicates were done, which are consist of four seed populations obtained from self-fertilization of independent parents. More than 100 seeds were used in each seed population. Radical protrusion was scored as germination at a 4-h interval during first 3 days, then daily until day 7.

### Laser Scanning Confocal Microscopy (LSCM)

To observe NaCl-induced autophagosome formation, 4- or 5-days-old vertically grown *GFP-AtATG8a* seedlings were immersed in 100 mM NaCl, or 0.5 μM ConA, or both NaCl and ConA, for 30 min and 1 h, respectively, and scanned with a Leica SP5 (Leica, Germany) with a same set of scanning parameters. For each condition, at least 30 seedlings from three to five biological replicates were imaged, and >30 root cortex cells in the root hair zone were quantified for the numbers of autophagosomes/autophagic bodies.

For imaging of Na^+^, 5-days-old Arabidopsis seedlings were transferred to liquid 1/2 MS (control) or 1/2 MS containing 100 mM NaCl (salt stress) for 6 h, and then CoroNa Green AM (Thermo Fisher Scientific) was added into the medium to a final concentration of 5 μM. Two hours later, the seedlings were stained with 5 μM FM4-64 (Thermo Fisher Scientific) for 5 min, washed thoroughly, and scanned with a SP5 (Leica, Germany) confocal microscope following previous reports (Meier et al., 2006; Oh et al., 2010). At least 20 seedlings from three biological replicates were imaged for each line in each condition, and >30 root cortex cells in the meristematic zone were quantified for the CoroNa Green AM fluorescent intensities in Image J.

### Gene Expression Analysis

Ten-day-old seedlings were transferred to liquid 1/2 MS medium containing 150 mM NaCl for 0 (control), 30 min, and 3 h. RNA extraction, reverse transcription, RT-PCR and q-RT-PCR were performed as described (Xiong et al., 2016). *EF1a* (At5g60390) was used as an internal control. Three biological replicates consisting of three technical repeats each were done. Transcript levels of the nine salt-inducible genes following NaCl treatment in *ATG8-OX, atg5*, and *atg9* were compared with both WT control and with the same germplasm control using Student’s *t*-test. Primers used are listed in **Supplementary Table S1**.

## Results

### NaCl Treatment Rapidly Induces Autophagy in Arabidopsis Seedling Root Cortex Cells

**FIGURE 1:**
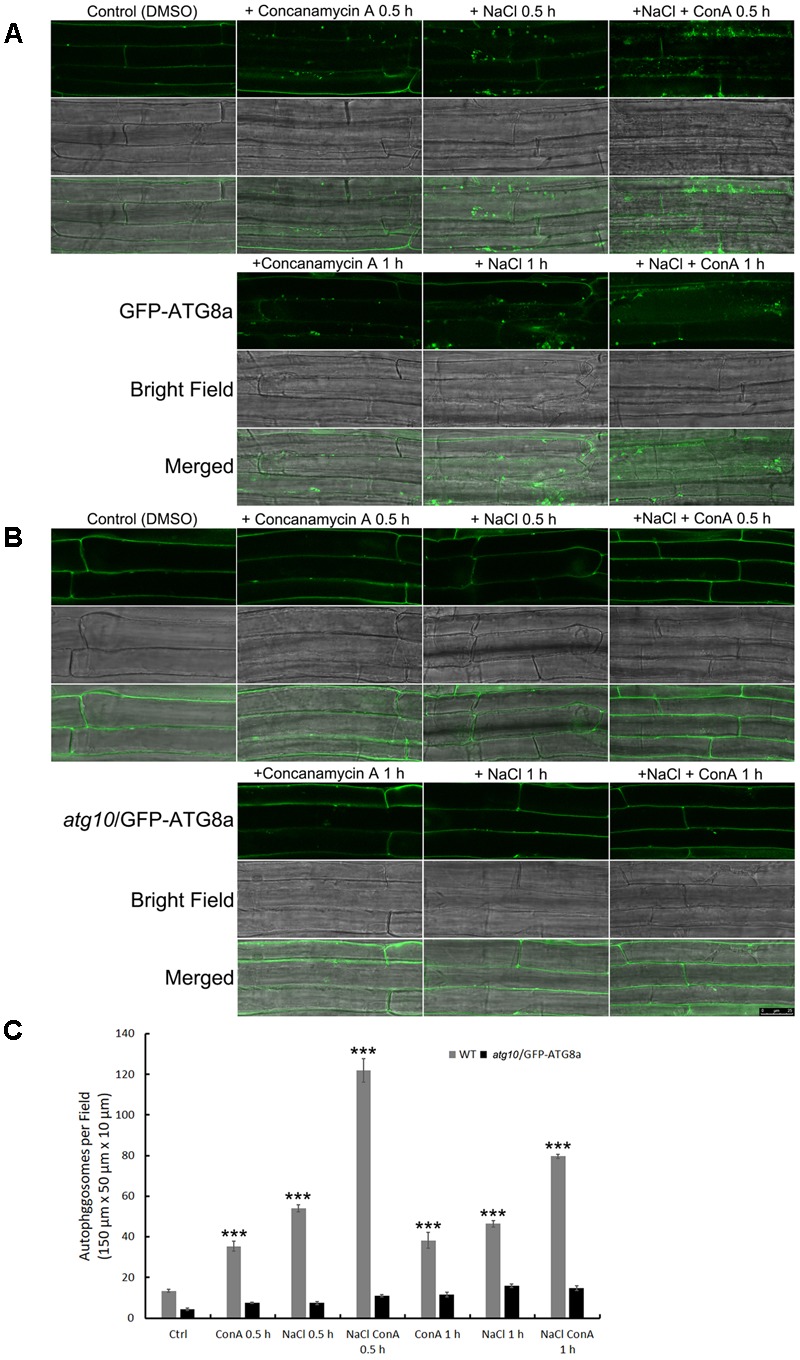
Autophagic flux is induced rapidly by NaCl treatment in Arabidopsis root cortex cells. **(A,B)** Sub-cellular distribution of the autophagosome marker GFP-AtATG8a in the root cortex cells was observed with confocal microscopy. **(A)** In the wild-type background, sporadic puncta representing autophagosomes were observed under controlled condition. Concanamycin A (ConA) treatment increased the number of autophagosomes. NaCl treatment (100 mM, 30 min) also increased the numbers of autophagosomes. NaCl plus ConA treatment further increased the number of autophagosomes and autophagic bodies. At 1 h of NaCl treatment, autophagosomes were still detected, but were less in numbers compared with the 30 min time point. **(B)** In *atg10* background, few autophagosomes were detected, and their numbers were not induced by ConA or NaCl. Bar = 25 μm. **(C)** The numbers of autophagosomes per field were quantified from >30 cells of 20 seedlings from at least three biological replicates. ^∗∗∗^*p* < 0.001. Bar = standard error.

To see if the autophagic flux is affected by salt stress, we firstly observed autophagosome formation and autophagic body turnover by looking at the autophagosome marker GFP-ATG8a in transgenic Arabidopsis seedlings carrying *ProATG8a:GFP-ATG8a* (**Supplementary Figure S2**). The marker line has a similar growth rate to the WT both under normal growth conditions and under nitrogen or carbon limitation, or salt and osmotic stresses (**Supplementary Figure S2**). The vacuolar proton pump inhibitor Concanamycin A (ConA) was used to preserve the autophagic bodies in the central vacuole. Under controlled conditions, GFP-ATG8a signals were mainly observed in the cytoplasm, with sporadic, relatively large (1–2 μm in diameter) puncta representing autophagosomes also detected (**Figure 1A**, DMSO). In the presence of ConA, additional smaller puncta representing autophagic bodies were observed in the vacuole (**Figure 1A**, +ConA 0.5 h). When treated with 100 mM NaCl for 30 min, large puncta accumulated (**Figure 1A**, +NaCl 0.5 h). In the presence of ConA, the numbers of autophagosomes and autophagic bodies increased even more, indicative of induced autophagic flux (**Figure 1A**, +NaCl +ConA 0.5 h). Interestingly, the number of ATG8a puncta was slightly reduced at 1 h of salt treatment (**Figure 1A**, lower panel), suggesting that the autophagic flux peaked shortly after salt stress. Quantification of the number of autophagic bodies confirmed that the autophagic flux, represented by the difference between the numbers of autophagic bodies treated with NaCl only and that of NaCl plus ConA treatment, peaked at 0.5 h (**Figure 1C**). Imaging of an *atg10* mutant carrying *GFP-ATG8a* under the same conditions showed that few autophagosome/autophagic bodies could be detected in the mutant with or without NaCl or NaCl plus ConA (**Figure 1B**). The imaging results indicated that autophagic flux can be rapidly induced by NaCl treatment in Arabidopsis.

### Autophagic Flux Is Elevated Shortly after NaCl Treatment in the Wild-Type

**FIGURE 2:**
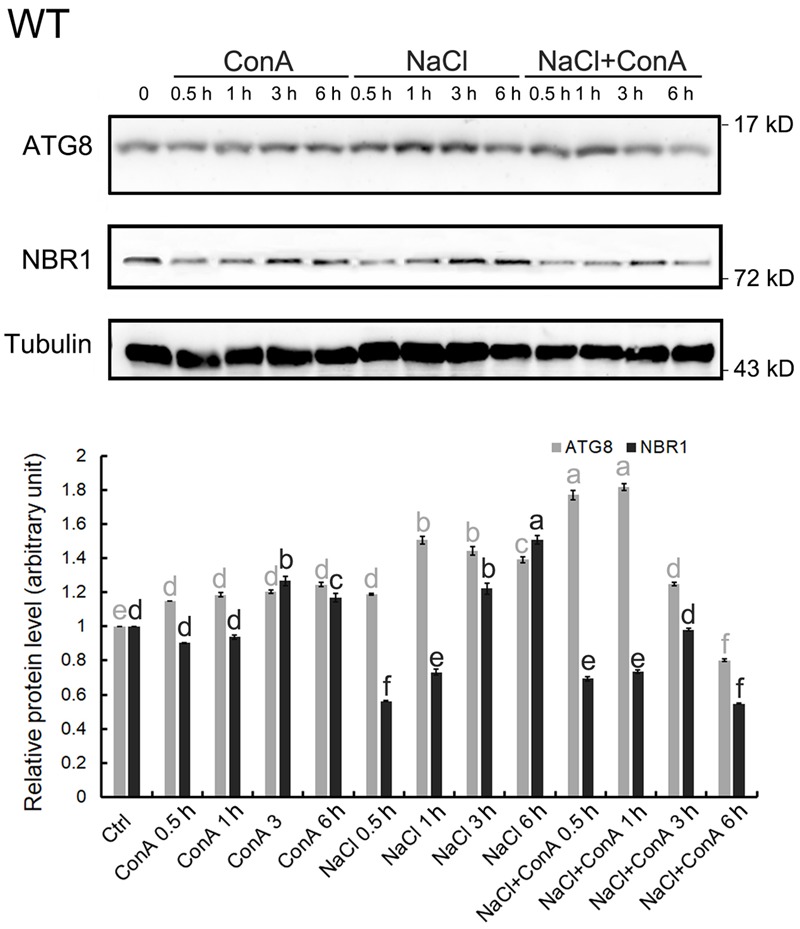
Autophagy is induced within 30 min of salt stress in the wild-type seedlings. The level of autophagy is represented by the difference in ATG8 protein levels between Concanamycin A (ConA)-treated and untreated samples at the same time points. In 150 mM NaCl-treated wild-type (WT) seedlings, induced autophagy can be observed mainly from 0.5 to 1 h upon stress. Selective autophagy, represented by differences in the amount of NBR1 in ConA+/-samples, can also be observed at 0.5 h. All SDS-PAGE gels contained 6 M urea. Anti-Tubulin antisera were used as internal control. Band intensity was quantified in Image J and normalized twice; firstly to Tubulin of the same time points, then to the controlled condition (time point 0), which was set as 1. Statistical analysis was performed by Tukey’s test. Columns labeled with the same letter are not significantly different (*p* ≤ 0.01). Bar = standard error.

To confirm that NaCl treatment can rapidly induce autophagy, immunoblotting of ATG8s was performed. We first examined whether the anti-GmATG8c antisera could detect both the lipidated and the non-lipidated ATG8s following a previous report (Suttangkakul et al., 2011). Total membrane fraction from the wild-type and *atg7* seedlings were collected, and the solubilized samples either treated or not with phospholipase D (PLD) were analyzed with immunoblotting. Unfortunately, the antibodies preferentially recognize un-lipidated ATG8s (**Supplementary Figure S3**). Hence the induction of autophagy was analyzed by comparing the difference in ATG8 protein levels between Concanamycin A (ConA)-treated and untreated samples at the same time points. Consistent with the imaging results (**Figure 1**), the levels of ATG8s were significantly induced at 0.5 and 1 h of NaCl plus ConA treatment (**Figure 2**).

Since the autophagic flux can also be detected by measuring NBR1 degradation (Mizushima and Yoshimori, 2007), changes in NBR1 levels following salt stress were monitored. The clear reduction in NBR1 protein level at 0.5 h of salt stress, in combination with the relative constant levels of NBR1 at the same time points in the presence of ConA (**Figure 2**), indicated that NBR1-dependent, selective autophagy peaked at 0.5 h following NaCl treatment.

Salt-induced ATG8 flux, represented by an elevation in ATG8 levels following NaCl treatment and an even higher induction in NaCl plus ConA treatment, was not observed in *atg5* or *atg7* mutants (**Supplementary Figure S4**). ATG8 levels were induced in *atg2* and *atg9* only at 0.5 h (**Supplementary Figure S4**). NBR1 degradation was observed at 0.5 and 1 h in *atg9*, and at 6 h in *atg2*, however, not in *atg5* or *atg7* (**Supplementary Figure S4**).

### Oxidized Protein Levels Are Transiently Reduced after NaCl Treatment in the Wild-Type

**FIGURE 3:**
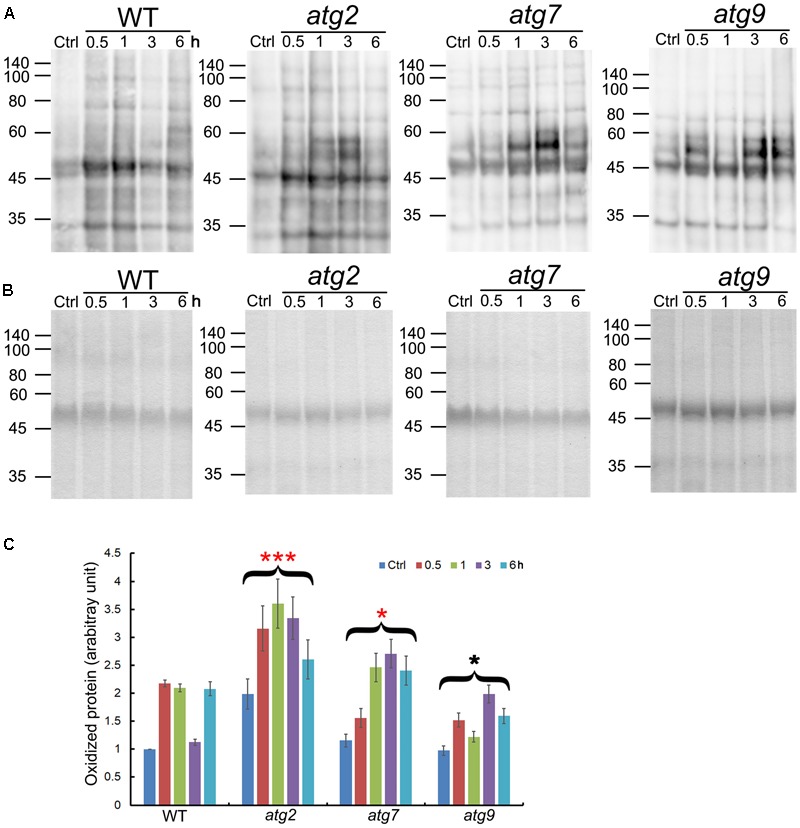
Quantification of oxidized proteins in WT and the autophagy mutants. **(A)** Ten-day-old WT, *atg*2, *atg7*, and *atg9* seedlings were transferred to liquid 1/2 MS containing 150 mM NaCl for the indicated time. Total proteins extracted were then derivatized by DNP, followed by immunoblotting with anti-DNP antibodies. Molecular mass (kDa) are indicated at the left. **(B)** Coomassie Blue staining of total proteins (before derivatization with DNP) as the loading control. Molecular mass (kDa) are indicated at the left. **(C)** DNP signals were quantified by densitometry from three independent repeats with the WT control value set as 1; ^∗∗∗^*p* < 0.001, ^∗^*p* < 0.05. Bar = standard error.

Oxidized proteins induced by ROS are known substrates for autophagy (Xiong et al., 2007). To see whether autophagy may contribute to the clearance of oxidized proteins generated upon salt stress, levels of oxidized proteins were analyzed over a time-course of 6 h. In the WT, oxidized proteins accumulated significantly within 0.5 h of salt treatment, got back to the control level at 3 h, before returning to the 0.5–1 h level by 6 h. *atg9* performed better than the WT in the clearance of oxidized proteins, with a reduction clearly observed at 1 h. *atg2* and *atg7* both had higher-than-WT levels of oxidized proteins before stress, which then got further induced by salt stress. Only at 6 h did the levels of oxidized proteins drop slightly in *atg2* and *atg7*. Overall, the results indicate that autophagy could have a positive role in transiently reducing the level of oxidized proteins generated by salt stress (**Figure 3**).

### The Autophagy Mutants Are Hypersensitive to Salt Stress during Germination

**FIGURE 4:**
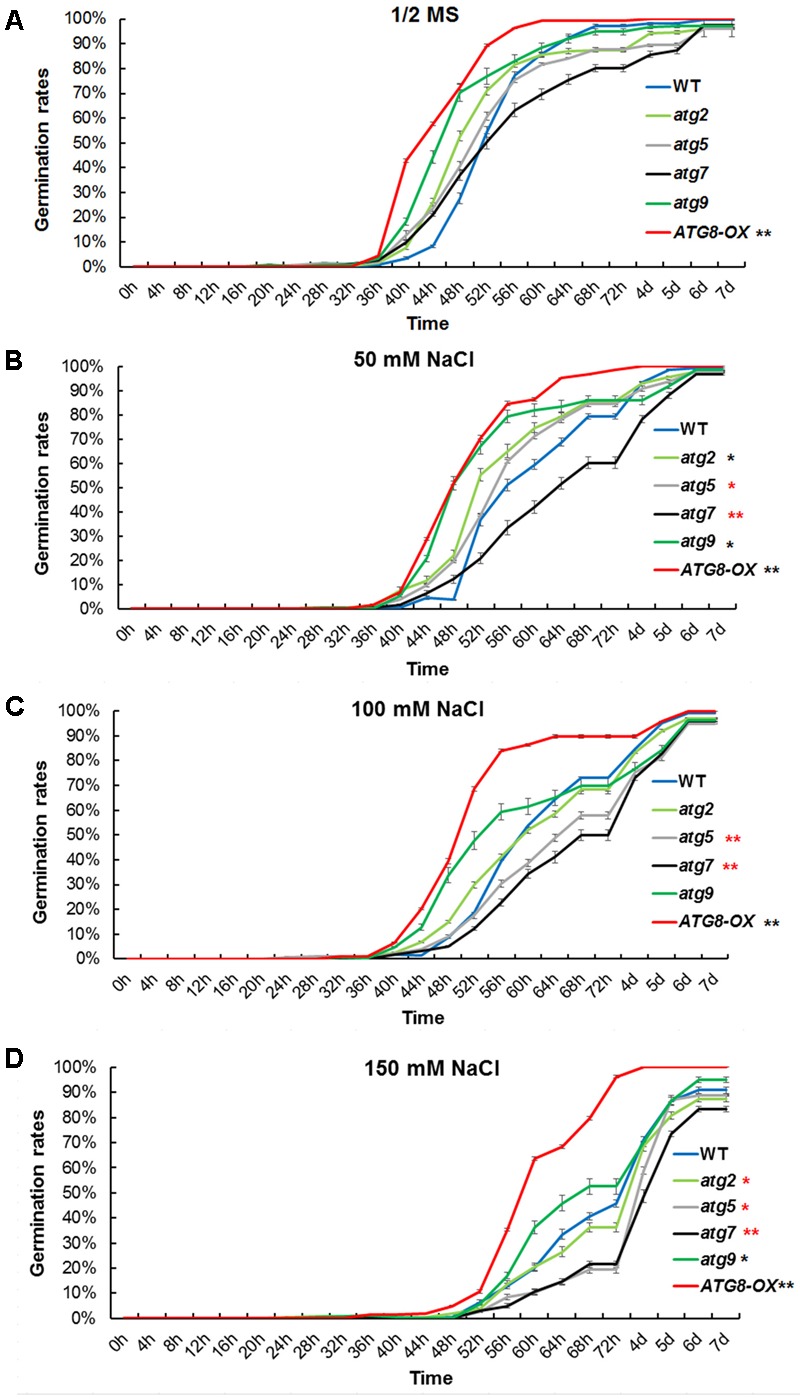
Germination time correlates with the level of autophagy upon salt stress. **(A)** Germination rates of *atg2, atg5, atg7*, and *atg9*, along with the wild-type and *Pro35S:GmATG8c (ATG8-OX)* on 1/2 MS were documented over 7 days (*n* > 200 each). **(B)** Germination rates on 1/2 MS containing 50 mM NaCl. **(C)** Germination rates on 100 mM NaCl. **(D)** Germination rates on 150 mM NaCl. ^∗^*p* < 0.05, ^∗∗^*p* < 0.01, respectively. Red star = slower than WT in germination; black star = faster than WT in germination. Bar = standard error in **(A–D)**.

To see if autophagy is positively involved in salt stress adaptation, germination percentage of the autophagy mutants and *ATG8-OX* plants were documented in a time course on control and NaCl-containing plates. Under controlled condition, *ATG8-OX* germinated significantly faster than the wild-type (*p* < 0.01, paired student’s *t*-test), whereas the autophagy mutants were statistically similar to the wild-type (**Figure 4A**). In the presence of NaCl, germination was slowed down in all lines, especially at higher concentrations (**Figures 4B–D**). The two autophagy-deficient lines, *atg5* and *atg7*, germinated significantly slower than the wild-type on all salt stress conditions (**Figures 4B–D**). *atg9* and *atg2* germinated faster than the wild-type on 50 mM NaCl, but not on higher concentrations (**Figures 4B–D**).

Similar germination curves were observed in the *atg* mutants on mannitol-containing plates (**Supplementary Figure S5**). These physiological data suggested that the level of autophagy is positively correlated with the germination rate at salt- and osmotic- stress conditions.

Consistent with the germination phenotypes, root-bending assay performed on 150 mM NaCl showed that the WT and the ATG8-OX lines responded normally to gravity stimulation on vertical plates supplemented with 150 mM NaCl. In contrast, *atg5* and *atg7* had less bending in their primary roots on NaCl plates (**Supplementary Figure S6**). Altogether, the phenotypes confirmed that autophagy is required for the adaptation of seedlings toward salt stress.

### Accumulation of Osmolytes Is Correlated with the Level of Autophagy upon Salt Stress

**FIGURE 5:**
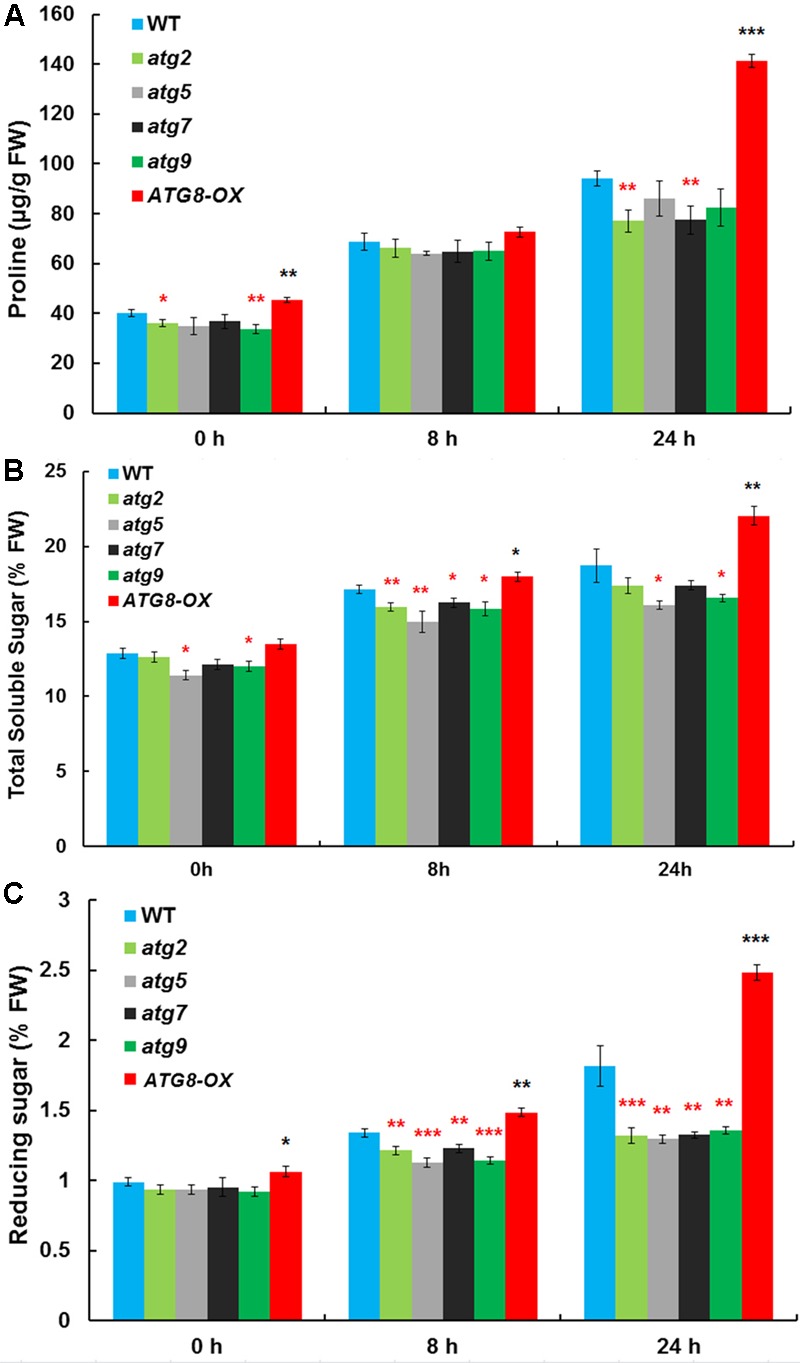
The autophagy mutants accumulate less compatible osmolytes upon salt stress. **(A)** Proline contents; **(B)** total soluble sugar contents; **(C)** reducing sugar contents in *atg2, atg5, atg7*, and *atg9*, along with the wild-type and *Pro35S:GmATG8c (ATG8-OX)* following 150 mM NaCl treatment. ^∗^*p* < 0.05, ^∗∗^*p* < 0.01, ^∗∗∗^*p* < 0.001, respectively. Red star = lower than WT; black star = higher than WT at the same time point. Averaged results from three biological replicates, each consisting of four technical repeats, are shown. Bar = standard error in **(A–C)**.

Autophagy may cater to the timely production of salt-induced osmolytes, such as proline and soluble sugars. The levels of proline, soluble sugar, and reducing sugar were measured in control and salt-stressed (0, 8, and 24 h) seedlings (**Figures 5A–C**). Before salt treatment, *ATG8-OX* had significantly higher levels of proline and reducing sugar than the wild-type, whereas several *atg* mutants had significantly lower levels of the osmolytes compared to the wild-type. Following salt treatment, all lines accumulated more osmolytes. *ATG8-OX* generally had much higher levels of proline and sugars, especially at 24 h (**Figures 5A–C**). In contrast, the *atg* mutants accumulated significant less osmolytes compared with the wild-type (**Figures 5A–C**), suggesting that the production of proline and soluble sugars indeed relies on autophagy.

### Expression of Salt-Inducible Genes Was Similar in Autophagy-Reduced and Autophagy-Enhanced Lines

**FIGURE 6:**
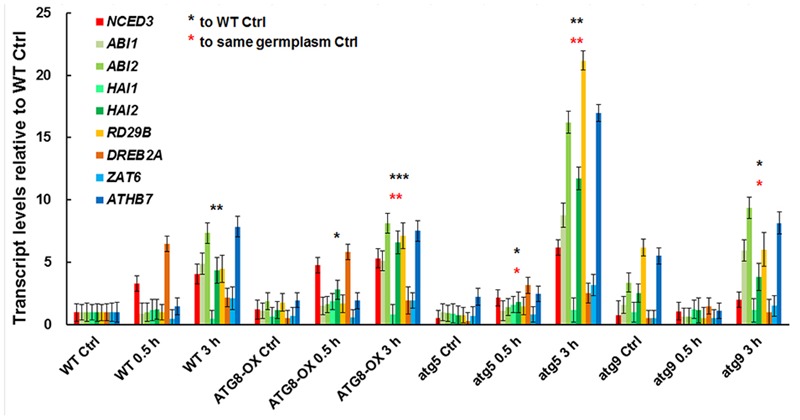
Expression of salt-inducible genes in WT, *ATG8-OX, atg5*, and *atg9* following salt treatment. Ten-day-old seedlings were incubated in liquid 1/2 MS containing 150 mM NaCl for 0 (control), 30 min, and 3 h. Transcript levels of selected salt-inducible genes were profiled and normalized to the wild-type control. ^∗^*p* < 0.05, ^∗∗^*p* < 0.01, ^∗∗∗^*p* < 0.001, respectively. Red star = compared with WT at time point 0; Black star = compared with the same germplasm at time point 0. Three biological replicates, each consisting of three technical repeats were done, and one representative replicate is shown. Bar = standard error.

To see if the salt-induced transcription is affected by the level of autophagy, we compared the expression patterns of salt-inducible genes in *atg5* and *atg9* mutants, *ATG8-OX*, and the wild-type with quantitative RT-PCR (**Figure 6**). The selected genes are known to be induced by salt stress either dependent or independent of ABA (Ma et al., 2006). Since we observed an induction of autophagic flux at 0.5 h of NaCl treatment, two relatively early time points (0.5 and 3 h) were selected for the transcript analysis. Expression of most markers was induced at 0.5 h in the wild-type, *atg9*, and *ATG8-OX*, and was further induced at 3 h (**Figure 6**). The expression patterns observed in the wild-type, *atg9*, and *ATG8-OX* indicated that autophagy may not affect salt-inducible gene expression directly. The extra high induction in gene expression observed in *atg5* (**Figure 6**), however, is unexpected, and might have resulted from a yet unknown regulatory mechanism.

### Sequestration of Sodium Ions in the Vacuole of Root Cells Is Correlated with Autophagy Levels

**FIGURE 7:**
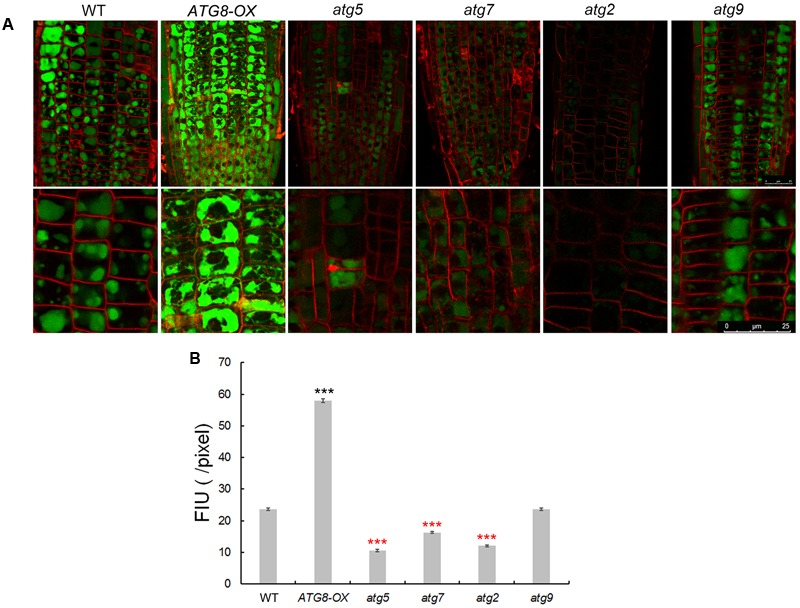
Imaging of Na^+^ in seedling roots of WT, autophagy mutants, and *ATG8-OX*. **(A)** Roots of 5-day-old wild-type, *atg5, atg7, atg2, atg9*, and *ATG8-OX* seedlings were incubated in liquid 1/2 MS containing 100 mM NaCl for 6 h, stained with CoroNa Green AM (5 μM) in the presence of 100 mM NaCl for 2 h, and scanned with a confocal microscope. The root tip region is shown (upper panel). Cortex cells in the meristematic region are 2.5× magnified (lower panel). Plasma membrane was stained with FM4-64 before scanning. **(B)** Quantification of CoroNa Green AM fluorescent intensity in **(A)**. More than 30 cells from more than 20 roots (three biological replicates), were quantified. Bar = 25 μm in **(A)**. ^∗∗∗^*p* < 0.001. Red star = lower than WT; black star = higher than WT in **(B)**.

To see whether autophagy might play a role in Na^+^ compartmentation upon salt stress, sodium ions were visualized with CoroNa Green, a fluorescent dye specific for Na^+^-imaging, with a same set of scanning parameters on a confocal microscope. The lipophilic dye FM4-64 was used to stain the PM, outlining the cells. Without NaCl, the fluorescence was barely visible (**Supplementary Figures 7, 8**) as reported (Oh et al., 2010). After 8 h of 100 mM NaCl treatment, striking differences in fluorescent intensity was observed among the lines (**Figure 7** and **Supplementary Figure S7**). As described (Oh et al., 2010), Na^+^ accumulated in the vacuoles of root cortex cells in all lines, however, at very different quantities. Compared with the wild-type, very weak signals were detected in *atg2, atg5*, and *atg7*, whereas much stronger signals were observed in *ATG8-OX* (**Figure 7** and **Supplementary Figure S8**). *atg9* had lower-than-WT yet clearly visible signals (**Figure 7** and **Supplementary Figure S9**). The observation suggested that sodium compartmentation in the root cortex cells positively correlates with the level of autophagy.

## Discussion

### *ProATG8a:GFP-ATG8a* as a New Marker Line for Autophagy

In the past decade, several methods have been established for observing autophagy in plants (Klionsky et al., 2016; Pu and Bassham, 2016), including staining acidic vesicles with the fluorescent dye monodansylcadaverine (MDC), electron microscopy of cells, etc. The most widely used method is to observe the presence and distribution of *Pro35S:GFP-ATG8s* and *ProUBQ10:GFP-ATG8s* in plant cells (Yoshimoto et al., 2004; Contento et al., 2005; Suttangkakul et al., 2011; Shin et al., 2014). Studies had revealed, however, that ectopic expression of ATG8s could have measurable impact on plant development. The *Pro35S:GFP-ATG8* plants generally grow faster to larger sizes (Slavikova et al., 2008; Xia et al., 2012). In this study, we constructed *GFP-ATG8a* driven by its own promoter, and generated T3 homozygous lines. Line 5-1 had phenotypes indistinguishable from the WT under normal growth conditions, and shared the same starvation- and stress- induced phenotypes with the WT. A bonus is that the T-DNA insertion site in this line had been revealed from TAIL-PCR, so that the marker line can be easily genotyped after crossing with other lines. We also noticed that in this line, the strong nuclear ATG8 signals commonly observed in both *Pro35S:GFP-ATG8* and *ProUBQ10:GFP-ATG8* under controlled conditions (Zhou et al., 2013; Pei et al., 2014) is absent (**Figure 1** and **Supplementary Figure S2**), indicating that the ATG8a level in this line is closer to the endogenous level.

### Autophagy Is Rapidly Induced by Salt Stress

An interesting finding in this study is that the autophagic flux peaks as early as 0.5 h upon salt stress in Arabidopsis seedlings (**Figures 1, 2**). Similarly, it has been reported that autophagic flux was induced at 30 min following hypertonic stress (350 and 500 mOsmol/kg NaCl, approximately 150 to 250 mM NaCl) on in LLC-PK_1_ renal proximal tubule-like cells (Nunes et al., 2013). Such speedy induction of autophagy is more likely based on post-translational modification rather than *de novo* synthesis of core autophagy proteins. What the possible modifications are remain to be explored. Apart from the possible regulation on ATG8 deacetylation (Huang et al., 2015), a known determinant on ATG8 activity, the redox-controlled ATG4 activity (Perez-Perez et al., 2014, 2016), might also regulate salt-induced autophagy. The ATG4 protease not only cleaves nascent ATG8 at its conserved C-terminal Glycine residue to promote conjugation of ATG8 to PE during autophagosome formation, but cleaves the amide bond between ATG8 and PE on completed autophagosome to recycle ATG8 (Yu et al., 2012). The two Arabidopsis ATG4s have been demonstrated to have different substrate preference *in vivo* and *in vitro* (Woo et al., 2014), and the activity of Chlamydomonas ATG4 has recently been shown to be inhibited by oxidation at a Cysteine residue (C400) conserved between plants and yeasts (Perez-Perez et al., 2016). It can be postulated that the oxidative stress generated during salt stress may modulate ATG8-PE formation by regulating ATG4 activity.

### Autophagy Is Likely Required for the Efficient Establishment of Salt Tolerance

The germination assay clearly indicates that autophagy plays a positive role in ensuring timely germination upon salt stress treatment. There are at least two explanations for such observation. Firstly, autophagy is known to contribute to nutrient remobilization from source to sink (Guiboileau et al., 2012; Xia et al., 2012), and autophagy mutants had insufficient protein degradation in their rosette leaves (Guiboileau et al., 2013). Therefore, during seed maturation, the acquisition of seed storage proteins, free amino acids, fatty acids, and other macromolecules is likely defective in the autophagy mutants (Galili et al., 2014). When challenged by salt stress, the germinating *atg* mutants could have insufficient osmolyte production, thus exhibiting sensitivity toward osmotic challenge. In contrast, the *ATG8-OX* seeds could have benefited from higher levels of storage proteins and other macromolecules during germination. Such differences become clearer as the stress conditions become more severe (**Figure 4** and **Supplementary Figure S5**), especially on the two autophagy deficient mutant, *atg5* and *atg7*. Indeed, a global analysis on etiolated (carbon starvation) autophagy mutant seedlings showed that, amino acids, organic acids, and protein levels were significantly decreased in *atg5* (Avin-Wittenberg et al., 2015). Secondly, autophagy participates in the clearance of damaged organelles such as peroxisomes (Kim et al., 2013; Shibata et al., 2013; Yoshimoto et al., 2014), and salt stress is known to generate such damages. Salt stress also leads to accumulation of protein aggregates and oxidized proteins (Mano et al., 2014), both of which have been reported to be substrates of autophagy (Toyooka et al., 2006; Xiong et al., 2007). On the other hand, autophagy is certainly not the only degradation pathway responsible for the elimination of damaged organlles, oxidized proteins, and protein aggregates. Our observation that the level of oxidized proteins are only transiently reduced in the wild-type indicated that autophagy may not be the major pathway involved in the long-term clearance of oxidized proteins. It has been reported that proteins oxidized in salt-stressed Arabidopsis are versatile not only in their functions but also in their sub-cellular localizations (Mano et al., 2014). A number of them are predicted to localize to the apoplast, the plasma membrane, and the nucleus, thus are likely to be degraded by other pathways. A recent report showed that in severely salt-stressed tomato roots (250 mM NaCl, 6 h), two of the three catalytic subunits, β2 and β5, of the proteasome are transiently modified to generate the stress proteasome (Kovacs et al., 2017). At 24 h, a normal proteasome profile reappeared. Such a transient apperance of the stress-induced active proteasome coincites with oxidized protein degradation (Kovacs et al., 2017) and is likely a parallel pathway to autophagy in short-term salt stress response.

**FIGURE 8:**
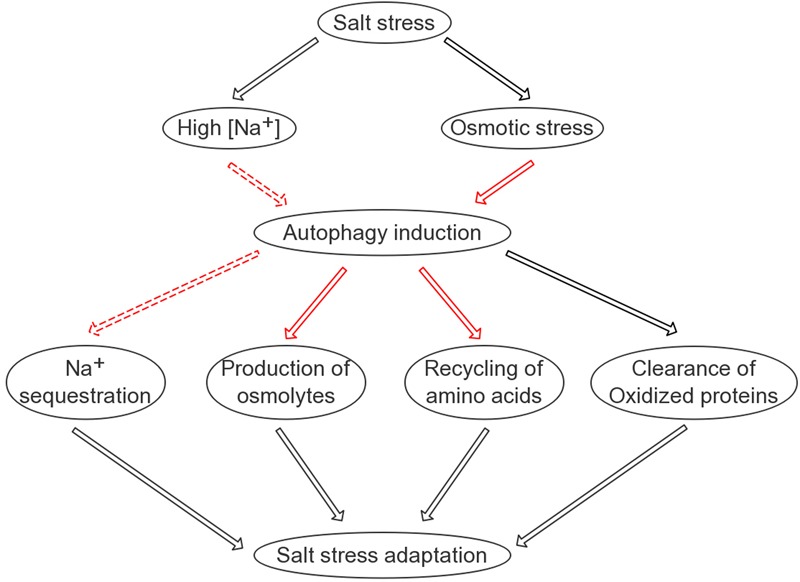
A simplified model on the possible roles of autophagy in salinity stress tolerance. Autophagy is positively involved in four aspects of plant adaptation to salt stress: sodium sequestration into the vacuole; elimination of oxidized proteins; recycling of free amino acids required for protein synthesis; and production of compatible osmolytes such as soluble sugars. Black lines indicate known pathways/regulations; solid red lines indicate relatively direct regulations identified in this study; dashed red lines indicate regulations identified in this study that are likely indirect.

Why does the autophagic flux, reflected by the turnover of both ATG8 and NBR1 in the lytic vacuole, take place at such an early time point? Considering that salt-induced transcription generally starts at a similar time point, it can be hypothesized that the efficient translation of stress-responsive proteins may rely on a reliable pool of amino acids. Indeed, the quantification of free amino acids following salt stress supported this hypothesis (**Supplementary Figure S9**). At the 8-h time point, the *ATG8-OX* seedlings had significantly lower levels of free amino acids, indicating that it was able to remobilize more amino acids for protein synthesis at an earlier time point. As a bulk degradation pathway, autophagy is likely a good candidate for maintaining such a pool. One may wonder which organelles or proteins are preferentially “eaten” during salt stress, since various substrates have been discovered in autophagy induced by different abiotic stresses. For instance, upon heat stress, autophagy is required for the clearance of the ubiquitinated protein aggregates (Zhou et al., 2013). Hypoxia-induced autophagy, on the other hand, may use chloroplast proteins as substrates, as the autophagy mutants die quickly following submergence and their detached leaves turn yellow faster upon ethanol treatment (Chen et al., 2015). Interestingly, autophagy also regulates the compartmentation of sodium ions in root cells, although the underlying mechanism remains uncovered. A model summarizing our findings is presented (**Figure 8**). The interesting fact that autophagy gets induced very rapidly following salt treatment and is required for successful salt adaptation may be explored in molecular breeding of salt tolerant crops in future.

## Author Contributions

QG, LL, and PZ conceived and designed the study. LL, PZ, DW, and QG analyzed the data and drafted the manuscript. LL, PZ, RZ, JF, JZ, JS, and ZW carried out the experiments. All authors have read and approved the final manuscript.

## Conflict of Interest Statement

The authors declare that the research was conducted in the absence of any commercial or financial relationships that could be construed as a potential conflict of interest.

## Footnotes

1 www.fao.org

2 http://rsb.info.nih.gov/ij/

## Supplementary Material

FIGURE S1 Illustration of materials used in this study.

FIGURE S2 *ProATG8a:GFP-ATG8a* as a marker line for autophagy.

FIGURE S3 The Anti-GmATG8c antisera preferentially detect the non-lipidated ATG8s.

FIGURE S4 Autophagy is not induced by salt treatment in autophagy mutants.

FIGURE S5 Germination of *atg* mutants, WT, and *ATG8-OX* on mannitol.

FIGURE S6 Root bending assay of *atg* mutants, WT, and *ATG8-OX* on 150 mM NaCl.

FIGURE S7 CoroNa Green staining of root tips of *ATG8-OX* lines.

FIGURE S8 CoroNa Green staining of root tips without NaCl.

FIGURE S9 Changes in free amino acid levels in lines with altered autophagy levels upon salt stress.

TABLE S1 Primers used in this study.
