# Supplementary material for: Autophagy Is Rapidly Induced by Salt Stress and Is Required for Salt Tolerance in Arabidopsis
Source: Front Plant Sci. 2017 Aug 22;8:1459. doi: 10.3389/fpls.2017.01459 (PMC5572379; doi:10.3389/fpls.2017.01459)
Supplement: FIGURE S1 — Illustration of materials used in this study. [file Presentation_1.pdf]

Supplementary Material

Autophagy is rapidly induced by salt stress and is required for salt tolerance in Arabidopsis

Liming Luo[1,†], Pingping Zhang[1,†], Ruihai Zhu[1], Jing Fu[1], Jing Su[1], Jing Zheng[1], Ziyue Wang[1], Dan Wang[1*], and Qingqiu Gong[1*]

Supplementary Table 1 Primers used in this study.
Supplementary Figure 1 Illustration of materials used in this study.
Supplementary Figure 2 *ProATG8a:GFP-ATG8a* as a marker line for autophagy.
Supplementary Figure 3 The Anti-GmATG8c antisera preferentially detect the non-lipidated ATG8s.
Supplementary Figure 4 Autophagy is not induced by salt treatment in autophagy mutants.
Supplementary Figure 5 Germination of *atg* mutants, WT, and *ATG8-OX* on mannitol.
Supplementary Figure 6 Root bending assay of *atg* mutants, WT, and *ATG8-OX* on 150 mM NaCl.
Supplementary Figure 7 CoroNa Green staining of root tips of *ATG8-OX* lines.
Supplementary Figure 8 CoroNa Green staining of root tips without NaCl.
Supplementary Figure 9 Changes in free amino acid levels in lines with altered autophagy levels upon salt stress.

Supplementary Table 1 Primers used in this study.

**Mutant Verification Primers**		
atg2-1	Salk076727F	GTGGGGCTCATAGCTTAGACC
	Salk076727R	TCGAGTGATTCTGTGGTTTCC
atg5-1	CS806267F	ATTTGCTATTTGTTTGGCACG
	CS806267R	TACCGTTCATGACAGAGGTCC
atg7	CS862226F	CAGCGTGATCTGTGAGAACTG
	CS862226R	TTCTTGGAGCTGGTACATTGG
atg9	CS874564F	AAAAGGTTCTTTCTTACGCCG
	CS874564R	CACATAATGCAACTCGTCGTG
atg10	Salk084434F	ACATAACCAATCGTTCCCTCC
	Salk084434R	ATCCTAAGACCAACCACCTGC
Salk left border	LBb1.3	ATTTTGCCGATTTCGGAAC
SAIL left border	LB3	TAGCATCTGAATTTCATAACCAATCTCGATACAC
**qRT-PCR Primers**		
Internal control	At5g60390EF1aF	AAGAGCGTGGACAAGAAGGA
	At5g60390EF1aR	AGTCTCATCATTTGGCACCC
Salt-regulated genes	ABI1At4g26080F	TGGCGAGTGACGGGGTTTGG
	ABI1At4g26080R	TCCGCTCATCCGCGAGCAAC
	ABI2At5g57050F	GTCGCGGCAAAACGCCACTC
	ABI2At5g57050R	CGAGCCCCGTTCCACCGGAT
	NCED3At3g14440F	AATGAACAGCCCGTCCGGCG
	NCED3At3g14440R	CACCGGCTCGTGAAGTGGGT
	RD29BAt5g52300F	CCGAAGTCGCCACGGTCCG
	RD29BAt5g52300R	CCGCCACTGCCTCCCAACTC
	ATHB7At2g46680F	GCGACGCAAAAGAAGACACA
	ATHB7At2g46680R	CTCTTCCGGTTTACGCCTCC
	DREB2AAt5g05410F	AATGGCGACGATGTGTTTGC
	DREB2AAt5g05410R	CTTTGTTGACTCTCGGGCCT
	HAI1At5g59220F	TAGACCGGATTCAAGCAGCG
	HAI1At5g59220R	AGGGACAAGAGTGTTTTCGTCA
	HAI2At1g07430F	AGAGGTTGTTCGTTGGGGTG
	HAI2At1g07430R	ATCCAACAGCGTCACAGTCC
	ZAT6At5g04340F	ACAAGGCAAGTCACCGGAAA
	ZAT6At5g04340R	CGACTTCACACTTCCTCCCC
**GFP-ATG8a Construct Primers**		
Promoter	pAtATG8a-GFPF	CCTCTAGAGTCGACCCCGACACAAGGTTAAGGGGCTCG

	pAtATG8a-GFPR	CTAGTCAGATCTACCATCGATCGTCTGCTAGATCGGGGGA
Gene	GFP-AtATG8aF	CACCACCACCACGTGATGATCTTTGCTTGCTTGAAATTCG
	GFP-AtATG8aR	GGGGAAATTCGAGCTTTATTCAAGCAACGGTAAGAGATCC

Supplementary Figure 1 Illustration of materials used in this study.

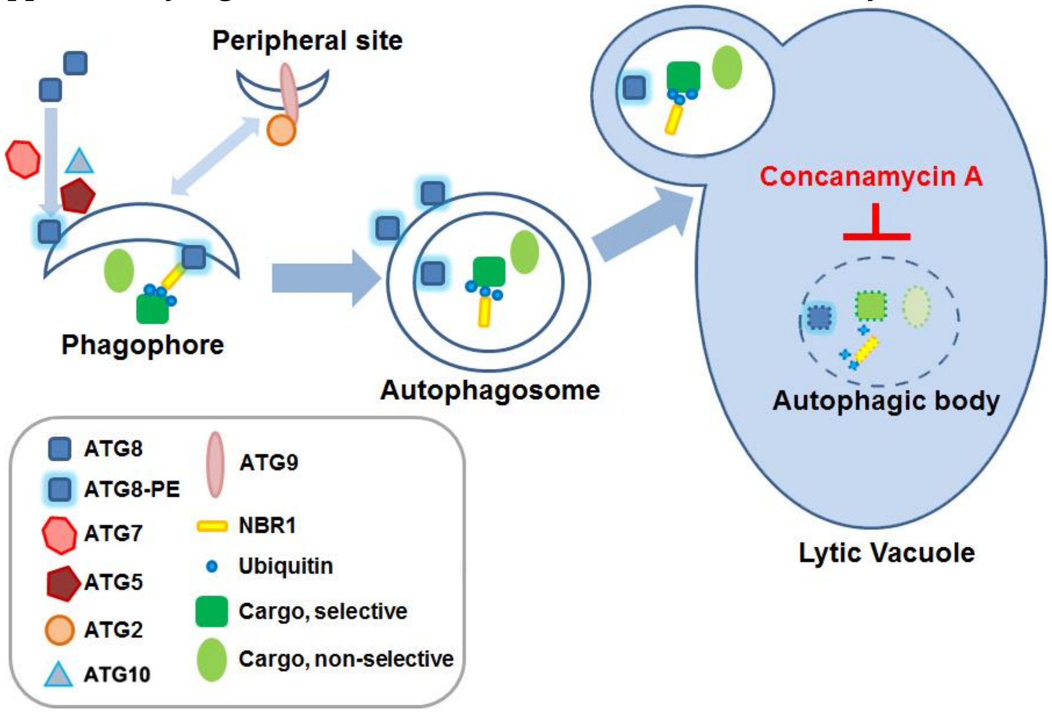

Null mutants for five of the core autophagy machinery genes- *atg5*, *atg7*, *atg10*, *atg2*, and *atg9*-are used along with the transgenic Arabidopsis over-expressing GmATG8c (*ATG8-OX*) for physiological, biochemical, and cell biology studies. ATG5, ATG7 and ATG10 are required for conjugation of ATG8 to phosphatidylethanolamine (PE). ATG5 also recruits ATG8 to the expanding phagophore. ATG2 mediates ATG9 cycling between an unknown membrane source and the phagophore. The incidence of autophagy is analyzed with immunoblotting of the autophagosome marker ATG8 and the autophagy receptor/adaptor NBR1, which binds both poly-ubiquitinated cargo and ATG8 in selective autophagy. To monitor autophagic flux, the vacuolar lumen alkalizer Concanamycin A (ConA) is used to preserve autophagic bodies in the lytic vacuole.

Supplementary Figure 2 *ProATG8a:GFP-ATG8a* as a marker line for autophagy.

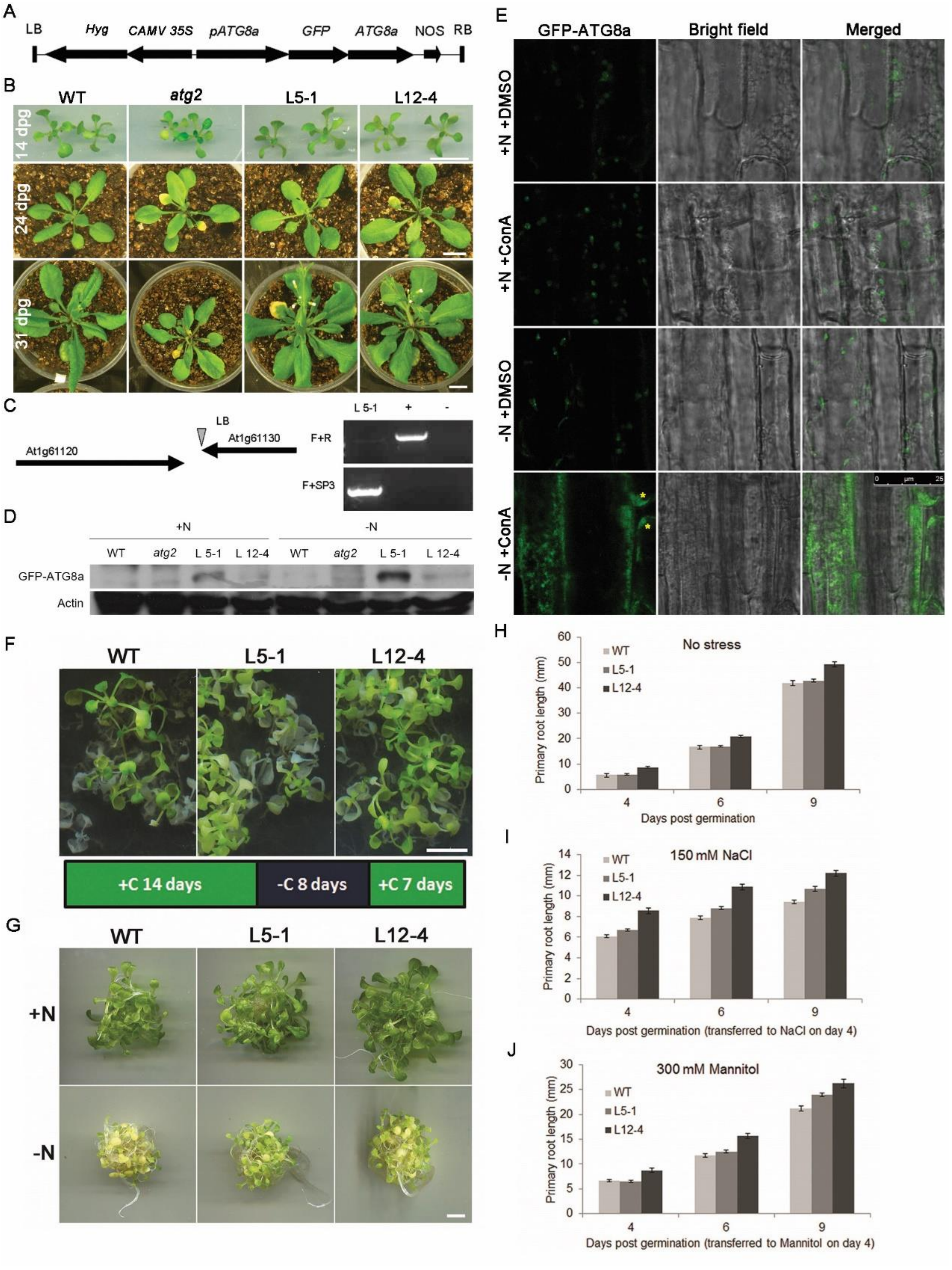

(A) Diagram of the construct. (B) Growth rates of *ProATG8a:GFP-ATG8a* T3 homozygous lines are similar to WT. *atg2* used as negative control. (C) T-DNA insertion site in L5-1. Left: The T-DNA is inserted at the end of *At1g61130*. Right: PCR verification of the insertion site. (D) Accumulation of GFP-ATG8a responds to nitrogen starvation in L5-1. Anti-GFP used to identify GFP-ATG8a. Anti-Actin used as internal control. (E) LSCM of GFP-ATG8a in L5-1 root epidermal cells. GFP-ATG8a can be observed at a minimum level at control condition (+N+DMSO),

and accumulates in the vacuole following concanamycin A treatment (+N+ConA). Upon nitrogen starvation, concanamycin A treatment preserve a relatively large amount of GFP-ATG8a in the vacuole (-N+ConA). (F-J) The marker lines are evaluated for their physiological responses towards carbon and nitrogen starvation, and salt and osmotic stresses. (F) Fourteen-day-old seedlings grown on 1/2MS plates under long-day condition were shifted to dark chambers for 8 days, and then let recover under long-day condition for 7 days. L5-1, L12-4 and WT have similar survival rates. (G) Ten-day-old, liquid-cultured seedlings of same counts, were transferred from 1/2 MS to fresh 1/2 MS (+N) or 1/2 MS without nitrogen (-N), and grown for a further 4 days. L5-1 and WT have similar biomass under both +N and -N conditions. (H) Four-day-old seedlings, vertically grown on 1/2 MS, were transferred to a new 1/2 MS plate and grown for a further 5 days. Primary root length was recorded on day 4, 6, and 9 post germination. (I) Seedlings same as in (H) were transferred to a 1/2 MS plate containing 150 mM NaCl, and primary root length recorded same as in (H). (J) Seedlings same as in (H) were transferred to a 1/2 MS plate containing 300 mM Mannitol, and primary root length recorded same as in (H). L5-1, L2-4, and WT had similar responses to salt and osmotic stresses (Student's t-test, $p>0.05$ in all cases.) Bar = 1 cm in (B), 25 μm in (E), 0.5 cm in (F) - (G). Bar = standard error in (H)-(J).

Supplementary Figure 3 The anti-GmATG8c antisera preferentially detect the non-lipidated ATG8s.

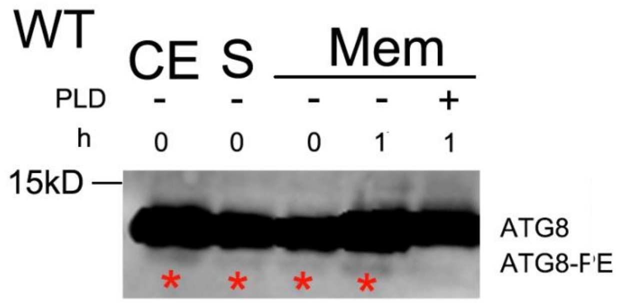

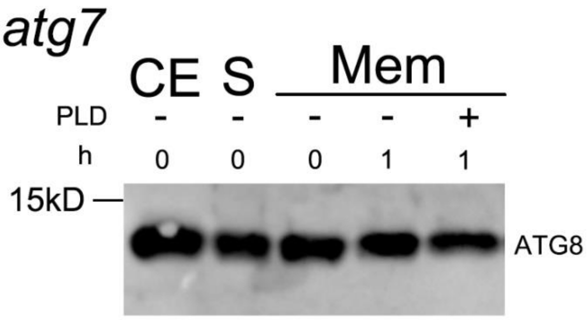

Ten-day-old vertically grown seedlings of wild-type and *atg7* were collected for protein extraction. Total membrane fraction (100,000 g, 1h) were collected, and the solubilized samples either treated or not with phospholipase D (PLD) were analyzed with immunoblotting. CE, crude seedling extract prior to fractionation; S, soluble fraction obtained from 100,000 g centrifugation; Mem, membrane fraction obtained from centrifugation and solubilized in Triton X-100. Fractions were treated with PLD at 37 °C for 1 h before separated on SDS-PAGE containing 6 M urea. Very faint bands representing ATG8-PE were detected in the WT samples without PLD treatment, but not in PLD-treated WT or in *atg7*. These low Mw bands (marked with *) likely represent ATG8-PE.

Supplementary Figure 4 Autophagy is not induced by salt treatment in autophagy mutants.

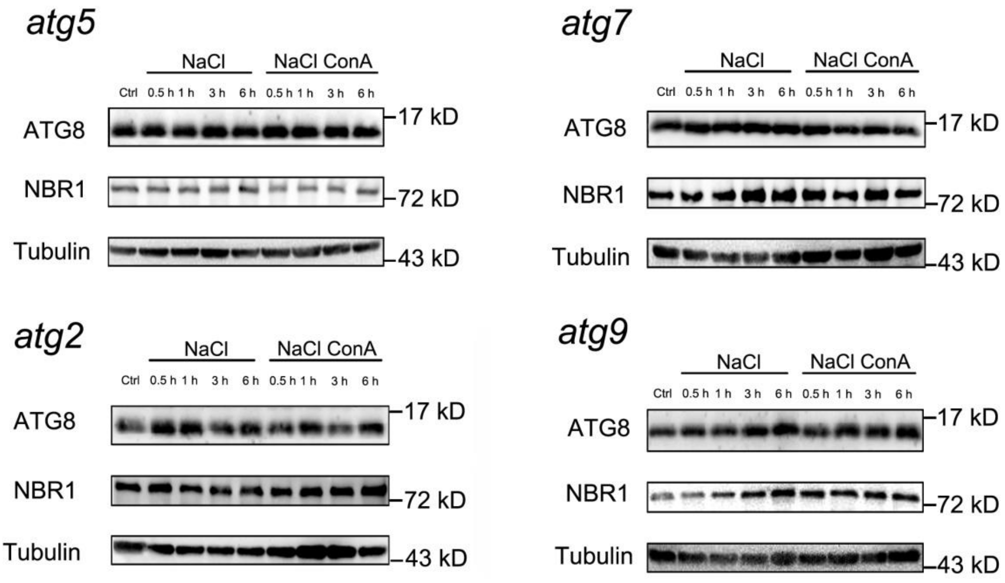

The level of autophagy is represented by comparing the ATG8 protein levels between Concanamycin A (ConA)-treated and untreated samples at the same time points. The level of selective autophagy is represented by differences in the amount of NBR1 in ConA+/-samples. All SDS-PAGE gels contained 6 M urea. Anti-Tubulin antisera were used as internal control. Only a mild reduction in NBR1 was observed in *atg9* at 0.5 h of NaCl treatment.

Supplementary Figure 5 Germination of *atg* mutants, WT, and *ATG8-OX* on mannitol.

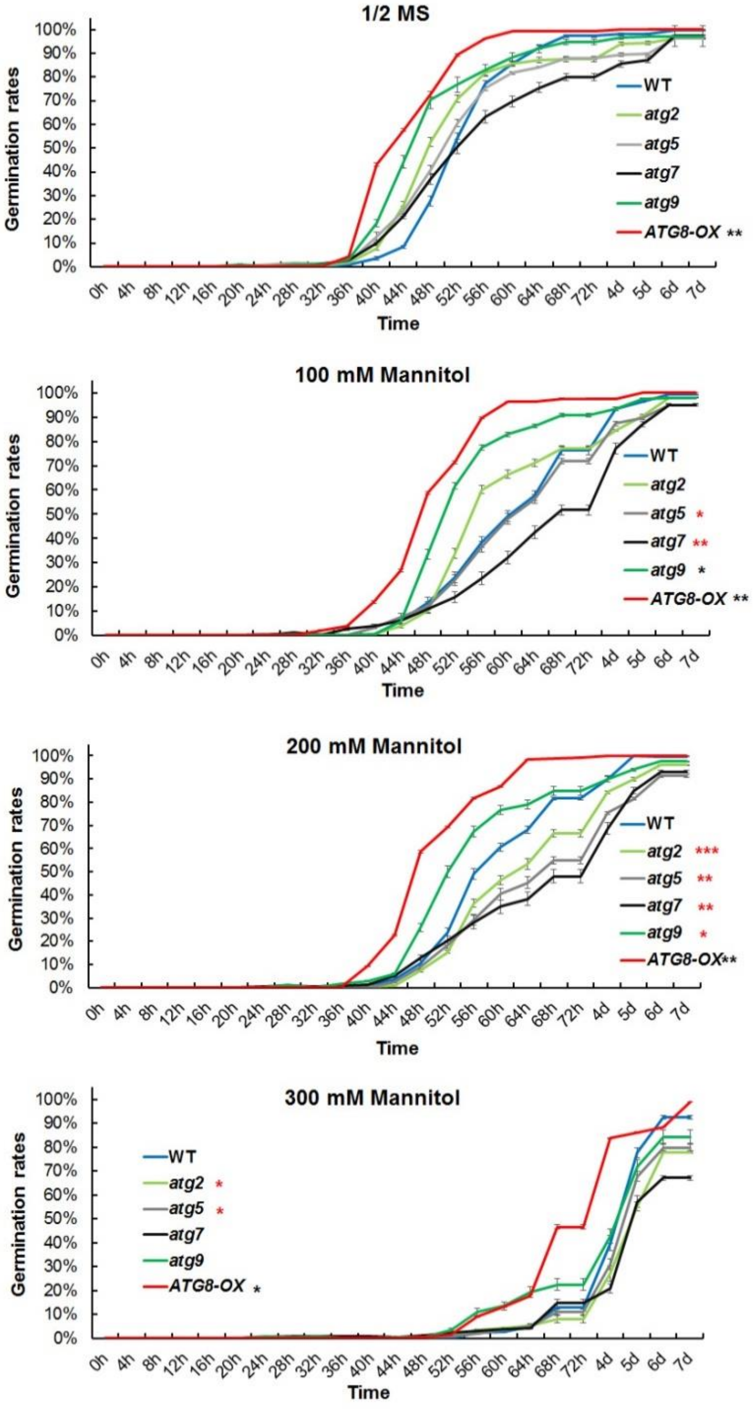

For germination assay on mannitol, stratified seeds were sown on 1/2 MS medium with or without mannitol. Germination rates were scored at a 4-hour interval during first 3 days, then daily until day 7. Four biological replicates were done (n > 200 each). Bar = standard error. *, **, and *** indicate p<0.05 and p<0.01, p<0.001, respectively. Red and black stars indicate hypersensitivity and reduced sensitivity compared with the WT.

Supplementary Figure 6 Root bending assay of *atg* mutants, WT, and *ATG8-OX* on 150 mM NaCl.

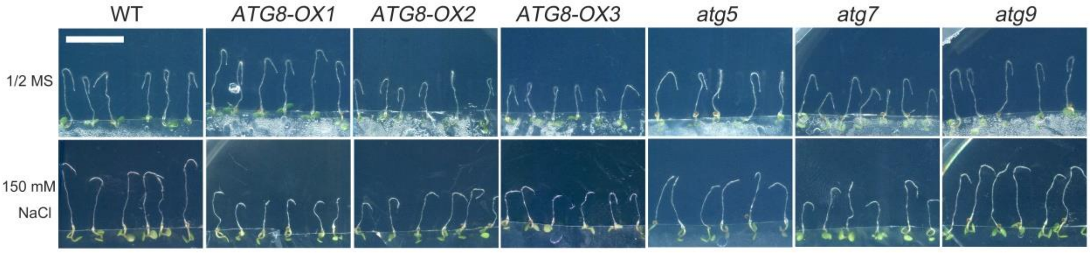

For root bending assay on NaCl, four-day-old vertically grown seedlings were transferred from 1/2 MS medium to 1/2 MS medium supplemented with 150 mM NaCl. To protect the cotyledons from bleaching, a sterilized plastic strip (8 mm wide) was placed on top of the medium, and the individual seedlings were placed with their cotyledons touching the strip only. The plates were then inverted. After 2 days, the plates were scanned. On 150 mM NaCl, insufficient root-bending was observed in *atg5* and *atg7*.

Supplementary Figure 7 CoroNa Green staining of root tips of in *ATG8-OX* lines.

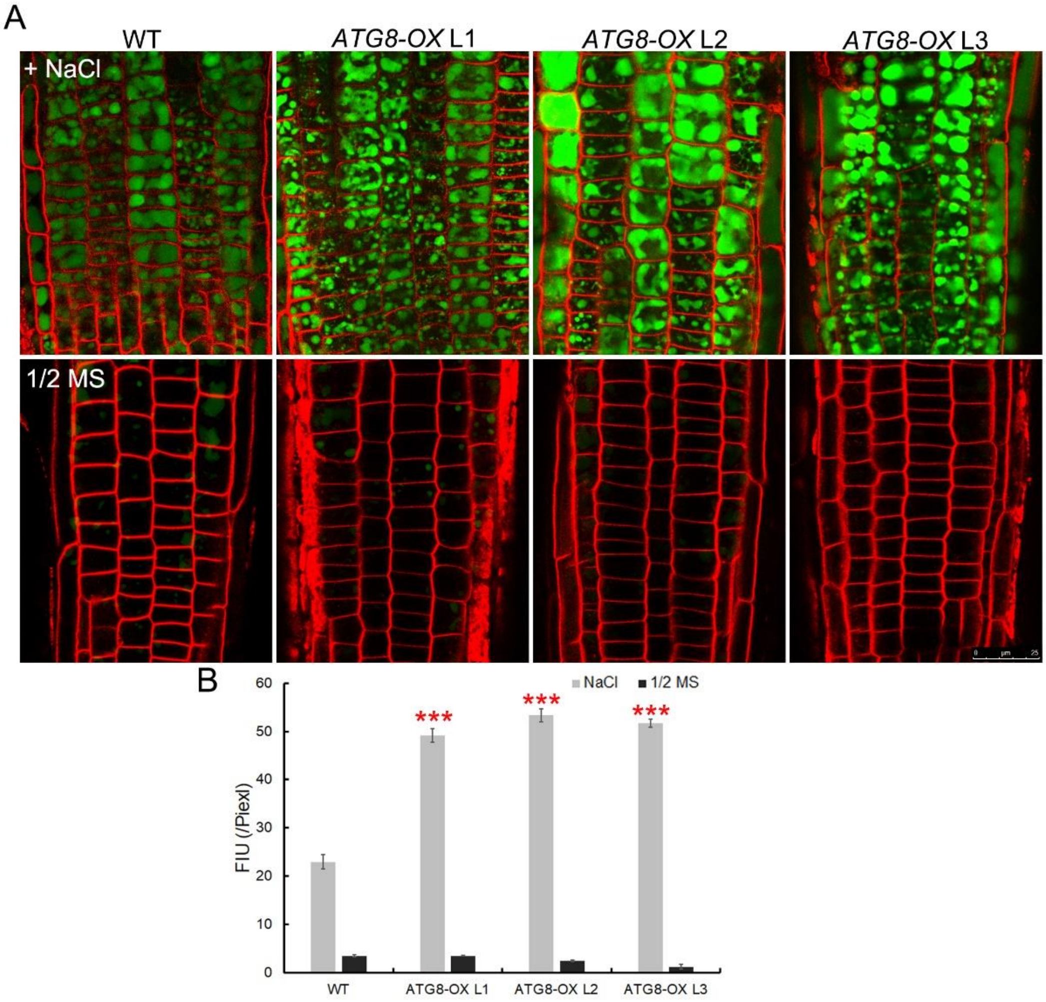

(A) Five-day-old three ***ATG8-OX* lines** seedlings incubated in liquid 1/2 MS

containing 100 mM NaCl for 6 hours, for 6 hours plus 2 hours of CoroNa Green AM (5 μM) (green) staining were scanned with a SP5 (Leica, Germany) confocal microscope as described (Meier et al., 2006; Oh et al., 2010). Plasma membrane was stained with FM4-64 (5 μM) (red) right before scanning. Confocal settings were completely same as for NaCl-treated seedlings shown in the manuscript. Bar = 25 μm. **(B)** Quantified Fluorescent intensity unit (FIU) of (A).

Supplementary Figure 8 CoroNa Green staining of root tips without NaCl.

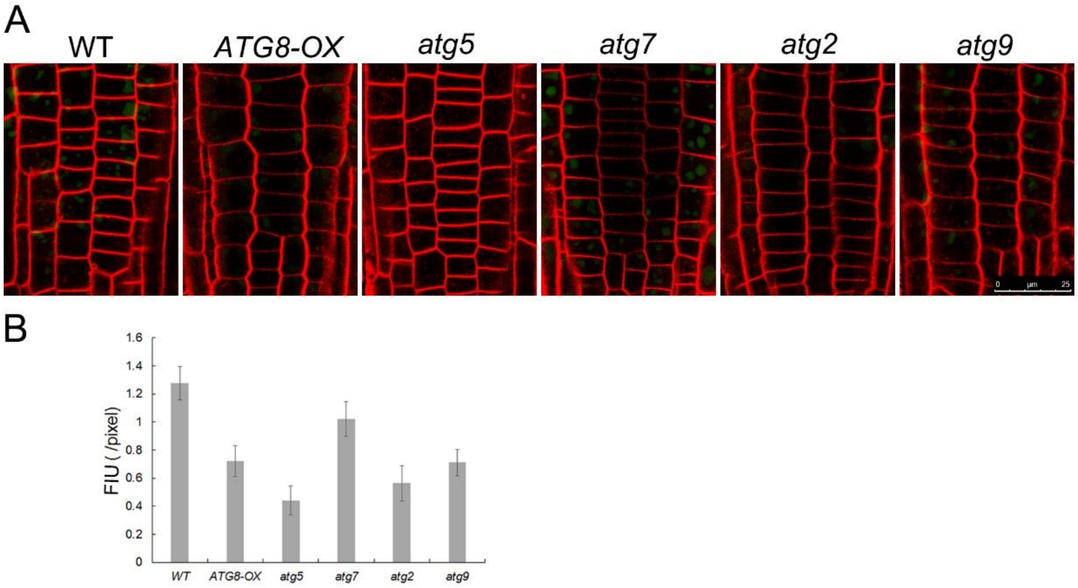

(A) Five-day-old seedlings incubated in liquid 1/2 MS for 6 hours plus 2 hours of CoroNa Green AM (5 μM) (green) staining were scanned with a SP5 (Leica, Germany) confocal microscope as described (Meier et al., 2006; Oh et al., 2010). Plasma membrane was stained with FM4-64 (5 μM) (red) right before scanning. Confocal settings were completely same as for NaCl-treated seedlings shown in the manuscript. Bar = 25 μm. **(B)** Quantified Fluorescent intensity unit (FIU) of (A).

Supplementary Figure 9 Changes in free amino acid levels in lines with altered autophagy levels upon salt stress.

A

ctrl	WT	*atg2* *	*atg5*	*atg7*	*atg9*	*ATG8-OX* *
Asp	0.0	0.6	1.6	0.4	0.0	0.6
Thr	-1.0	0.3	-1.0	-0.4	-0.9	-0.4
Ser	2.0	1.9	-0.7	2.0	1.6	3.0
Asn	3.4	5.3	2.5	4.6	3.5	4.0
Glu	4.8	5.4	1.6	5.5	4.8	5.3
Gly	1.9	0.8	-1.0	0.2	1.9	2.4
Ala	0.7	4.7	0.6	2.1	0.7	1.7
Val	-1.6	0.6	1.5	-0.2	-1.7	-0.5
(Cys)2	-1.9	-0.8	-1.1	-1.2	-1.9	-1.9
Met	-3.4	-2.2	-0.7	-2.6	-3.5	-2.5
Ile	-3.3	-1.9	-1.0	-3.2	-3.0	-2.8
Leu	-3.1	-1.4	-1.0	-2.5	-2.9	-2.5
Tyr	-4.9	-2.4	-2.5	-4.0	-5.0	-3.7
Phe	-3.0	-0.9	-0.7	-2.4	-3.0	-2.1
His	-0.3	0.7	1.2	1.1	-0.3	0.5
Lys	-1.4	-0.6	-0.7	-0.5	-1.5	-1.4
Arg	1.8	3.3	2.3	3.4	1.8	2.2
Pro	-0.8	-0.4	0.4	-0.3	-0.8	-0.3

Color scale: -6 0 6

B

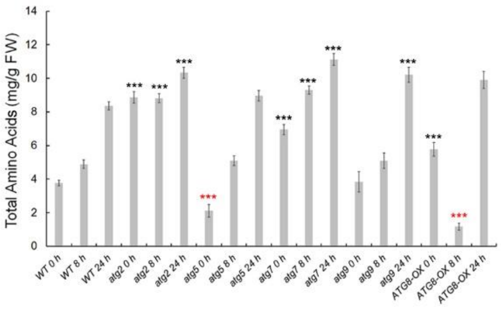

C

	WT 8 h	WT 24 h	*atg2* 8 h	*atg2* 24 h	*atg5* 8 h	*atg5* 24 h	*atg7* 8 h	*atg7* 24 h	*atg9* 8 h	*atg9* 24 h	*ATG8-OX* 8 h	*ATG8-OX* 24 h
Asp	0.4	0.9	-0.8	0.2	-0.9	-0.8	-1.0	0.5	0.3	0.7	1.0	0.2
Thr	0.4	1.0	-0.3	0.1	0.3	1.1	0.3	1.8	0.6	1.3	0.9	0.5
Ser	0.7	1.3	-1.0	0.4	2.7	3.7	-0.4	1.1	-1.0	0.6	0.0	-0.1
Asn	0.8	1.4	-0.7	-0.2	1.5	2.3	0.1	1.6	0.7	1.8	0.7	1.2
Glu	0.2	0.9	0.7	0.5	3.7	4.2	0.6	2.1	0.5	1.1	-1.7	0.6
Gly	0.2	0.7	-0.2	0.4	1.2	2.2	0.2	1.7	0.4	-0.6	-0.8	-1.2
Ala	0.2	1.8	-1.3	-0.3	0.7	2.3	2.1	3.6	-0.2	3.9	1.9	2.3
Val	0.0	1.9	-0.3	0.0	-2.1	-0.7	0.7	2.2	1.0	2.5	0.8	1.3
(Cys)2	-1.6	1.0	0.2	-0.1	-0.2	1.2	0.4	1.9	0.4	1.0	1.2	1.1
Met	-3.2	0.7	-0.1	-0.2	-2.4	-1.1	0.2	1.7	0.2	0.5	-0.7	0.1
Ile	-0.6	0.8	-0.1	-0.2	-1.8	-0.4	1.0	2.5	0.8	1.2	-0.8	0.9
Leu	-0.1	1.1	-0.1	-0.2	-1.1	0.4	1.3	2.8	1.2	1.6	0.5	1.2
Tyr	-0.1	1.1	-0.1	0.0	-1.2	1.0	1.5	3.0	1.2	1.5	0.6	0.8
Phe	0.6	1.2	-0.9	-1.1	-1.6	0.2	0.6	2.1	0.5	1.0	0.7	0.4
His	0.5	1.4	1.9	2.1	-1.0	0.4	-0.3	1.1	0.6	1.3	1.9	0.6
Lys	0.5	1.0	-0.3	0.5	-0.8	0.6	-0.4	1.0	0.2	1.2	2.0	1.1
Arg	0.5	1.6	-0.6	0.3	-0.7	1.1	-0.5	1.0	0.1	1.8	0.9	1.3
Pro	-0.3	1.9	-0.8	0.4	-1.0	0.9	-1.3	0.2	0.0	0.8	2.0	1.0

Color scale: -4 0 4

Ten-day-old *atg* mutants, WT, and *ATG8-OX* seedlings treated with 1/2MS containing 150 mM NaCl for 0, 8, and 24 hours were analyzed for their free amino acid contents using an amino acid analyzer. **(A)** Log2 values of amino acid contents (mg/g fresh weight) at controlled condition (time point 0) are shown as a heat map. **(B)** Total amino acid contents in the lines. (**C)** Log2 values of the fold changes in amino acid content at 8 and 24 hours relative to control are shown as a heat map. * indicates p < 0.05, *** indicates p <0.001 in **(A)-(B)**.
